# Modulated calcium-sensing receptor (CaSR) expression in human breast cancer provided insights into tumor progression and therapeutic potential

**DOI:** 10.1186/s43046-026-00338-x

**Published:** 2026-01-19

**Authors:** Omnia Mansour, Safaa M. Ali, Amani Kazem, Abeer El Wakil

**Affiliations:** 1 Department of Biological and Geological Sciences, Faculty of Education, Alexandria University, Alexandria, Egypt; 2 Department of Nucleic Acid Research, Genetic Engineering and Biotechnology Research Institute, City of Scientific Research and Technological Applications, Alexandria, Egypt; 3 Department of Pathology, Medical Research Institute, Alexandria University, Alexandria, Egypt

**Keywords:** HOTAIR, p38 MAPK, DSG1, DSC1, Caveolin-1

## Abstract

The calcium-sensing receptor (CaSR) plays a crucial role in cellular signaling and has been implicated in cancer progression. However, its specific role in breast cancer remains unclear. This study aimed to investigate CaSR gene expression levels, its correlation with oncogenic pathways, and its protein localization in human breast cancer tissues.

Quantitative real-time PCR (qRT-PCR) was used to analyze the relative expression of CaSR and associated genes in malignant and adjacent non-malignant breast cancer tissue samples. Pearson correlation analysis was performed to assess relationships between CaSR and other oncogenic markers. Immunohistochemistry (IHC) was conducted to evaluate CaSR protein localization and intensity in different breast cancer grades and fibroadenoma tissues.

Our findings revealed that 56% of patients exhibited elevated CaSR expression, with significant positive correlations between CaSR and oncogenic genes such as HOTAIR, SNOR78, BC200, DSG1, DSC1, HER2, BRCA1, p38 MAPK, and Akt (*p* = 0.001 for most correlations). Additionally, CaSR expression negatively correlated with menopausal status (*p* = 0.001), suggesting hormonal influences on its regulation. IHC analysis showed strong membranous and cytoplasmic staining in grade II tumors without metastasis (+ 3) but weak or undetectable CaSR expression in advanced metastatic cases. These findings indicate a potential role for CaSR in tumor progression, adhesion, and metastasis.

Importantly, this study provides novel evidence linking CaSR expression to non-coding RNAs, desmosomal adhesion molecules, and MAPK/AKT signaling pathways, highlighting previously unexplored mechanisms through which CaSR may influence breast cancer progression and metastatic behavior. Further research is needed to determine its potential as a biomarker or therapeutic target in breast cancer treatment.

**Supplementary Information:**

The online version contains supplementary material available at 10.1186/s43046-026-00338-x.

## Introduction

CaSR, a member of the class C G-protein-coupled receptor (GPCR) superfamily, plays a crucial role in maintaining calcium homeostasis and regulating intracellular Ca²⁺ signaling pathways. Calcium functions as a key second messenger, influencing essential biological processes such as gene transcription, cell cycle regulation, differentiation, migration, and apoptosis [1–4]. Additionally, it contributes to cell membrane stability, enzyme activation, muscle function, bone homeostasis, and blood clotting [3].

CaSR is expressed on the cell surface as a homodimer, stabilized by direct interactions between its extracellular (ECD) and transmembrane domains (TMD). The ECDs of both monomers interact in a side-by-side configuration via a covalent disulfide bond involving Cys-129 and Cys-131, while the TMDs establish hydrophobic interactions [5]. Beyond its role in calcium homeostasis, CaSR is widely expressed in tissues such as the central nervous system, cardiovascular system, lungs, gastrointestinal tract, pancreatic islets, adipose tissue, and skin [3, 4, 6–8]. Moreover, CaSR-mediated hormonal regulation integrates a network of interactions across multiple tissues, including the parathyroid glands, intestines, kidneys, and bone, as well as vitamin D metabolism [9, 10]. Additionally, CaSR can function in a paracrine manner, influencing neighboring CaSR receptors through its sensitivity to bicarbonate (HCO₃⁻), as observed in the colon [11, 12].

CaSR interacts with a diverse array of proteins that modulate its signaling profile. Among these, several CaSR-binding proteins have been identified, including inwardly rectifying potassium channels [13], filamin A, and receptor activity-modifying proteins (RAMPs) [4]. Additionally, CaSR can form heterodimers with other class C GPCRs, such as mGlu1a, mGlu5, and GABA_B receptors, as observed in both endogenous and recombinant systems [5].

Dysfunction of the CaSR is associated with mutations not only in calcium-related diseases but also in a wide range of conditions, including myocardial infarction, asthma, inflammation, pancreatitis, diabetes mellitus, Alzheimer’s disease, and cancer [1, 3, 4, 6, 7]. In cancer, the role of CaSR remains controversial, as it has been reported to function as both an oncogene and a tumor suppressor [3, 4]. Mutations in CaSR are also implicated in several non-calciotropic disorders. For example, the R990G variant has been linked to an increased risk of hypercalciuria and nephrolithiasis [14]. Additionally, alterations in CaSR activity or expression have been associated with variations in cardiac function, insulin secretion, postprandial blood glucose regulation, lipolysis, and the inhibition of myocardial cell proliferation [5].

Thus, the present study aims to investigate the expression levels of CaSR in female Egyptian patients diagnosed with invasive ductal carcinoma (IDC) grades II and III, comparing tumor tissues with their corresponding normal breast cells. Furthermore, we seek to explore its relationship with selected long noncoding RNAs (MALAT1, HOTAIR, and BC200) and small nuclear RNAs (SNOR76, SNOR78, and SNOR113-1) expression levels, as well as with key proteins involved in cell junctions [E-cadherin, desmoglein-1 **(**DSG1), and desmocollin 1 (DSC1)], endocytosis [Clathrin, caveolin-1 (Cav-1), and dynamin 1 and 3 (DYN1 and DYN3)], and cancer progression using qRT-PCR.

## Materials and methods

### Patients and the collection of specimens

This retrospective cohort study included 70 female breast cancer patients who underwent curative mastectomy at the Medical Research Institute, Alexandria University, Egypt. All patients were diagnosed with IDC at grade II or III. Clinicopathological data (summarized in Table S1) were retrieved from the pathology department’s databases and medical records, as detailed in our previous studies [15, 16]. The study protocol was approved by the Research Ethics Council of Alexandria University, Egypt (Approval No. E/C. S/N. R10/2023), following the ethical guidelines of the Medical Research Institute. Ethical considerations ensured that all procedures complied with institutional and international standards for research involving human subjects.

Following surgical removal and clinical evaluation, malignant breast cancer specimens and fresh non-malignant control samples were collected from each patient at the Clinical Pathology Department, Medical Research Institute, Alexandria University, Egypt. Including both malignant and non-malignant tissues established a strong foundation for comparative analyses, enhancing the reliability of the findings.

### Expression levels of the selected genes influence breast cancer progression and development

#### RNA isolation

Fresh samples were stored at -80 °C in RNA-later (Ambion, UK) before analysis. Total RNA was isolated from 30 mg of frozen tumor and control tissues using the ISOLATE II RNA Mini Column Kit (Applied Biochemistry, Egypt), following the manufacturer’s guidelines. The concentration and purity of RNA were evaluated using a NanoDrop spectrophotometer. Only samples with an A260/A280 ratio between 1.8 and 2.0 were deemed suitable for analysis, while those outside this range were considered contaminated. The extracted RNA was subsequently preserved at -80 °C until further use.

#### Quantitative Real-Time PCR (qRT-PCR) technique

The reaction setup and data analysis followed the guidelines of the Thermo PikoReal™ Real-Time PCR system, utilizing the SensiFAST™ SYBR^®^ No-ROX One-Step Kit (Bioline, UK) and specific primers. Forward and reverse primers (Qiagen, Germany) were designed to target gene expression for CaSR (Forward primer: 5’-GCT GTT TAT CTC CTC TAT G-3’, Reverse primer: 5’-GGG CTC TTT CCT ATT CAT-3’) and β-actin (Forward primer: 5’-GCT GTC ACC TTC ACC GTT-3’, Reverse primer: 5’-CTC ATC TGG CCT CGC TGT-3’) as a reference gene.

The qRT-PCR program included an initial cDNA synthesis step at 50 °C for 15 min. Reactions were conducted in a 20 µL mixture containing 10 µL of SensiFAST™ SYBR^®^ No-ROX One-Step Mix (1X), 0.2 µL of reverse transcriptase, 0.4 µL of RiboSafe RNase Inhibitor, 1.6 µL of forward and reverse primers (10 pm), 4 µL of RNA template (10 ng), and sterile water to complete the volume to 16 µL.

Thermal cycling conditions consisted of reverse transcription at 45 °C for 10 min, followed by polymerase activation at 95 °C for 2 min. This was followed by 40 cycles of 95 °C for 5 s (denaturation), 60 °C for 10 s (annealing), and 72 °C for 5 s (extension).

To ensure accuracy, all reactions were performed in duplicate. Relative gene expression was determined using the comparative Ct method (2^(-ΔΔCT)), with β-actin serving as the endogenous control and gene expression levels in non-malignant samples used as the reference calibrator.

#### Immunohistochemical staining technique

The immunohistochemical (IHC) technique was employed to evaluate CaSR protein expression in tumor samples, including fibroadenoma tissues (used as controls, *n* = 3) and human breast cancer tissues (*n* = 3 per tumor grade).

The IHC procedure involved the following steps: Serial 5 μm thick paraffin sections were mounted on coated glass slides, dewaxed in xylene, and gradually rehydrated through descending concentrations of ethanol before being rinsed with distilled water. To block nonspecific antibody binding, the slides were treated with 3% hydrogen peroxide (H₂O₂). Antigen retrieval was performed, followed by cooling and two washes in phosphate-buffered saline (PBS).

To minimize background staining, each section was incubated with 100–400 µL of Ultra V Blocking solution for 1 h at room temperature. Next, the slides were incubated overnight at 4 °C in a dark humidity chamber with a CaSR polyclonal antibody (catalog number ABP56765, Abbkine, China). Subsequently, the sections were treated with biotinylated goat anti-polyvalent antibody for 10 min, followed by incubation with streptavidin-conjugated horseradish peroxidase for an additional 10 min. After washing in PBS for 5 min, the reaction product was visualized using 3,3′-Diaminobenzidine (DAB), applied for 5–15 min in the dark at room temperature. All sections were counterstained with hematoxylin, dehydrated through a graded ethanol series, cleared in xylene, and mounted for microscopic examination. Images were captured using a digital camera.

CaSR expression was quantified using a six-point scoring system, where 0 indicated absent expression, 1 represented rare positive cells, 2 corresponded to non-uniform weak expression, 3 denoted non-uniform weak or intense expression, 4 indicated intense non-uniform expression, and 5 reflected strong uniform expression.

### Statistical analysis

Version 20.0 of the IBM SPSS software program (Armonk, NY: IBM Corp.) was used to perform all statistical analyses. Quantitative data were presented as the mean ± standard error of the mean (SEM). Group correlations were assessed using Spearman’s rank correlation coefficient test, with statistical significance set at *p* < 0.05.

## Results

### The relative fold change in gene expression levels

In this study, we analyzed the relative transcription expression levels of CaSR gene and other associated genes in malignant and adjacent non-malignant human breast cancer tissue samples. Gene expression levels were quantified using the qRT-PCR method.

Our findings revealed that 56% of the patients exhibited elevated CaSR gene expression, with a mean expression level of 4.94. Pearson correlation analysis demonstrated a significant positive correlation between CaSR expression and several genes, including HOTAIR (*p* = 0.016), SNOR78 (*p* = 0.001), BC200, DSG1, DSC1, HER2, BRCA1, p38 MAPK, and Akt (*p* = 0.001 for all correlations). These findings align with previous studies indicating that CaSR may play a role in breast cancer progression through its interaction with oncogenic and regulatory genes (Table 1).

**Table 1:** Correlation of relative fold change in expression levels of selected genes

	CaSR	
	Pearson Correlation	Significance (2-tailed)
E-cadherin	0.468	0.243
DSG1	0.986^**^	0.001
DSC1	0.987^**^	0.001
Clathrin	-0.016	0.971
Cav-1	0.398	0.329
DYN1	0.011	0.980
DYN2	-0.016	0.970
DYN3	-0.130	0.781
β-catenin	-0.042	0.922
Beclin 1	0.540	0.167
ERα	-0.037	0.931
HER2	0.970^**^	0.001
BRCA1	0.984^**^	0.001
p38 MAPK	0.987^**^	0.001
Akt	0.974^**^	0.001
MALAT1	-0.190	0.653
HOTAIR	0.804^*^	0.016
BC200	0.974^**^	0.001
SNOR76	-0.243	0.562
SNOR78	0.980^**^	0.001
SNOR113-1	-0.266	0.611

**. Correlation is significant at the 0.01 level (2-tailed)

*. Correlation is significant at the 0.05 level (2-tailed)

### Associations between CaSR and clinicopathological features in female breast cancer patients

**Fig. 1:**
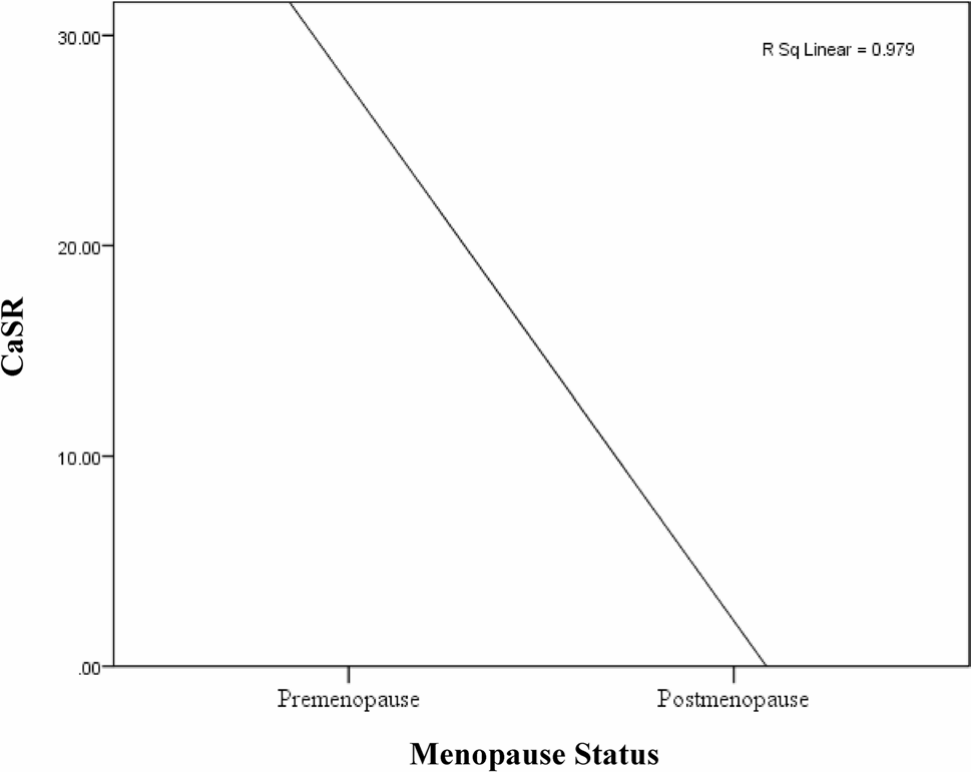
Scatter Plots Illustrating Significant Correlations Between Relative Fold Changes in Expression Levels of Gene Pairs Included in the Study

Additionally, a strong negative correlation was observed between CaSR expression and menopausal status (*p* = 0.001), suggesting that menopausal hormonal changes may influence CaSR-related gene expression in breast cancer patients (Fig. 1).

### IHC localization and intensity of CaSR in human breast cancer tissues

**Fig. 2:**
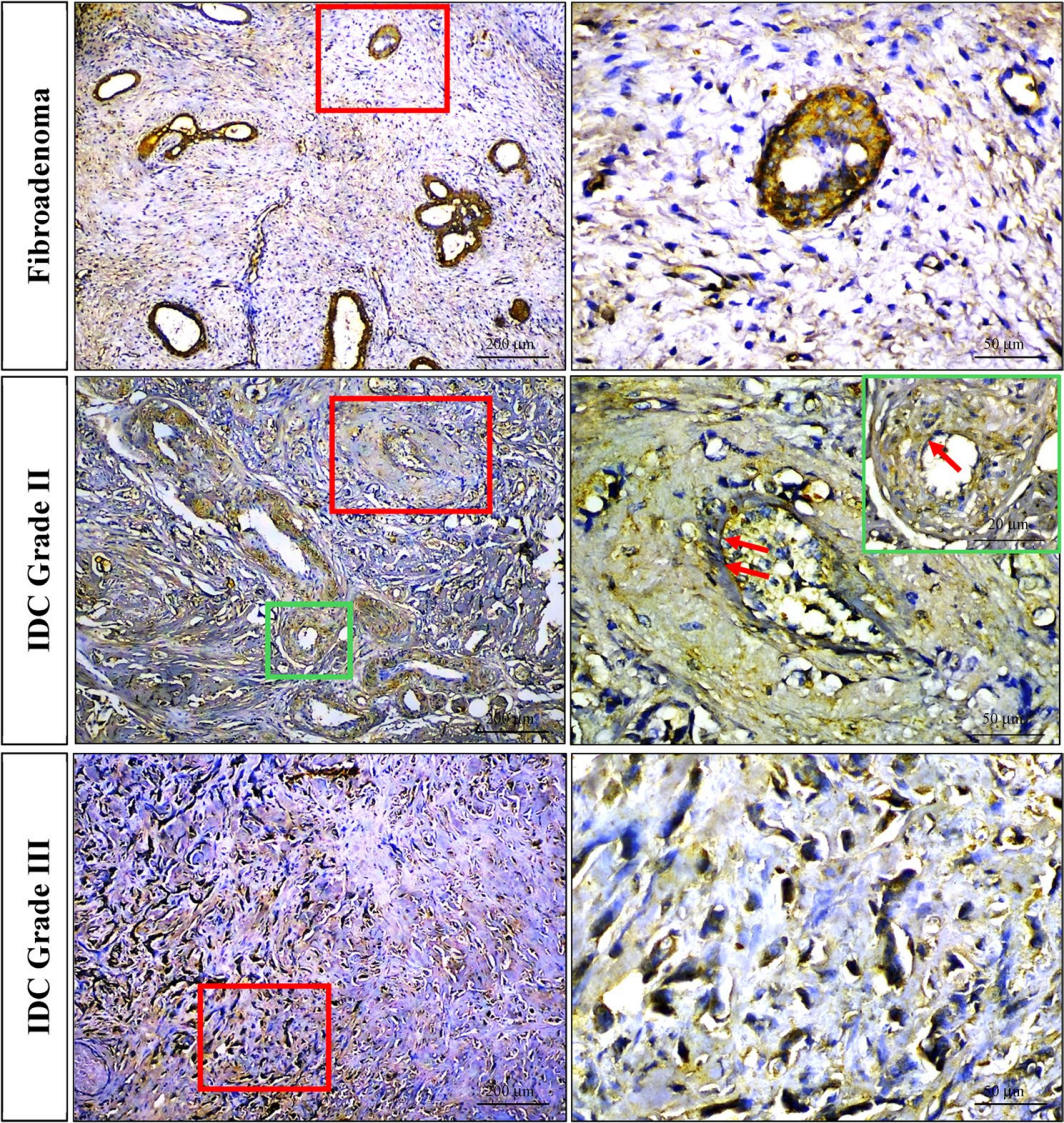
Representative photomicrographs of CaSR immunohistochemical staining in human breast tissue samples. The left column shows low-power views (magnification = 100×; scale bar = 200 μm), with corresponding high-power magnifications (400×; scale bar = 50 μm) of the delineated red squares shown in the right column. The delineated green square in the left column is magnified within the right column (scale bar = 20 μm). Fibroadenoma displays moderate cytoplasmic staining intensity (+ 2), while invasive ductal carcinoma (IDC) grades II and III exhibit weak cytoplasmic staining (+ 1). The red arrows highlight membranous staining in grade II IDC

To further investigate CaSR protein expression, we utilized an IHC approach to assess its localization and intensity in fibroadenoma and human breast cancer tissues (Fig. 2).

In fibroadenoma tissues, CaSR exhibited moderate cytoplasmic staining (+ 2), indicating a notable presence of the protein in these benign breast tumors. While in grade II breast cancer cases, patients with positive lymph node invasion and metastases exhibited weak cytoplasmic staining (+ 1). In contrast, those with negative lymph node invasion and metastases showed strong membranous and cytoplasmic diffusion (+ 3), suggesting a potential link between CaSR localization and metastatic behavior.

**Fig. 3:**
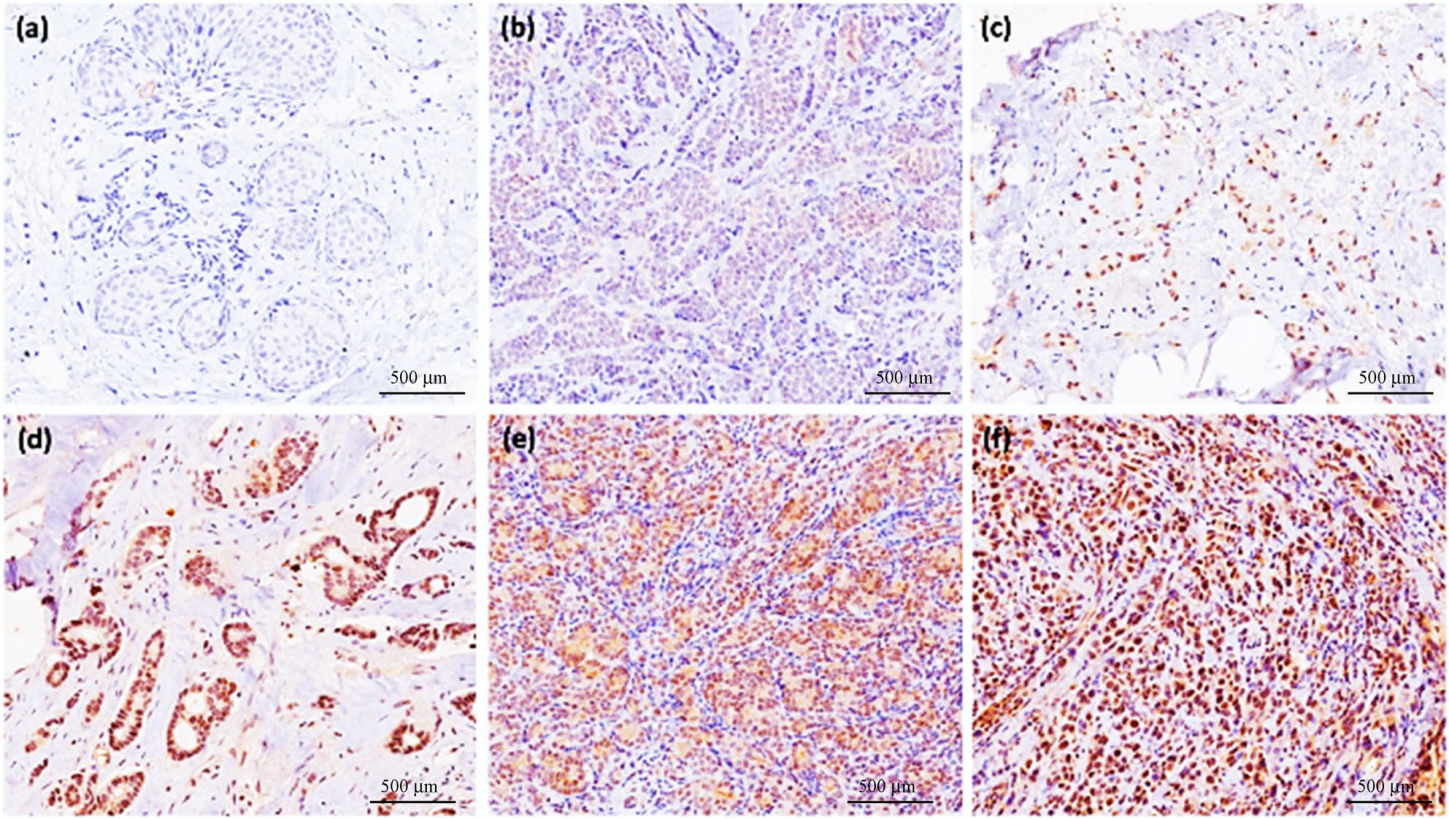
Immunohistochemical analysis of CaSR expression in breast cancer tissues. Staining intensity scores are illustrated as follows: **a **no expression (score 0); **b **rare positive cells (score 1); **c **non-uniform weak expression (score 2); **d **mixed weak and intense non-uniform expression (score 3); **e ** non-uniform intense expression (score 4); and **(f)** strong, uniform expression (score 5). Magnification = 20×, scale bar = 500 μm

In grade III breast cancer cases, patients with positive metastases but negative lymph node invasion showed no detectable CaSR staining. Among patients with both positive metastases and lymph node invasion (28 out of 34 cases), faint cytoplasmic staining (+ 1) was observed, reflecting a downregulation of CaSR expression and/or altered subcellular localization, with a shift toward the nucleus in advanced malignancy (Fig. 3).

These findings highlight the complex role of CaSR in breast cancer progression and its potential interaction with key oncogenic pathways. Further research is warranted to explore its implications in tumor aggressiveness, metastasis, and therapeutic targeting.

## Discussion

### The multifaceted role of CaSR in cancer and breast cancer progression

CaSR is a critical mediator of cellular processes that extend far beyond Ca²⁺ homeostasis. It is involved in gene transcription, cell cycle regulation, differentiation, migration, and apoptosis [1–4]. Furthermore, CaSR exhibits a wide range of functions across different tissue types and plays a pivotal role in hormonal regulation networks throughout the body [3, 4, 8]. Given its diverse biological roles, it is not surprising that CaSR dysfunction has been implicated in multiple diseases, including various types of cancer [6, 7].

### The dual role of CaSR in cancer: oncogene or tumor suppressor?

The role of CaSR in cancer remains controversial and highly context-dependent, with studies indicating that it can function either as an oncogene or a tumor suppressor, depending on the cancer type and its molecular environment [3, 4]. For instance, CaSR has been shown to exert tumor-suppressive effects in neuroblastomas, parathyroid cancer, and colorectal cancer, where its activation is associated with reduced proliferation and enhanced apoptosis. Conversely, in malignancies such as ovarian, prostate, and testicular cancers, CaSR appears to drive oncogenic processes, promoting cell survival, proliferation, and metastasis [2].

In maternal breast tissue, CaSR plays a critical role in regulating calcium homeostasis during lactation. However, in breast cancer, its function is more ambiguous, it behaves as an oncoprotein with compelling evidence suggesting its involvement in tumor progression and metastatic dissemination. Specifically, CaSR has been implicated in skeletal metastases, although the underlying mechanisms remain unclear [2]. Interestingly, some studies propose that CaSR activation may reduce breast cancer cell dissemination [17], while others report that low CaSR expression correlates with aggressive breast cancer phenotypes and increased metastasis rates [18], highlighting the receptor’s dualistic nature.

In our study, 56% of patients exhibited upregulated CaSR expression, with a mean expression level of 4.94. Based on our findings, we propose that CaSR does not function exclusively as a tumor promotor or suppressor in breast cancer. Instead, its role is likely dictated by prevalent cellular pathways and the tumor microenvironment. Whether CaSR promotes or inhibits tumor progression likely depends on the interplay between intrinsic cellular factors, extracellular cues, and the specific molecular landscape of the tumor. Further research is needed to elucidate these mechanisms, which could pave the way for CaSR-targeted therapeutic strategies in breast cancer management.

### Correlation between CaSR gene expression and clinicopathological features in breast cancer

An analysis of the clinicopathological characteristics of breast cancer patients in relation to CaSR gene expression revealed a statistically significant negative correlation with menopausal status (*p* = 0.001). This finding suggests that postmenopausal patients may exhibit lower levels of CaSR expression compared to premenopausal individuals. Notably, Busic-Pavlek et al. [2] identified additional significant correlations between CaSR expression and tumor size, lymph node invasion, and metastasis, further underscoring the potential involvement of CaSR in breast cancer progression.

Regarding estrogen receptor (ER) expression, the relationship with CaSR remains ambiguous. Wei [19] suggested that ER-positive breast cancers may exhibit varying levels of CaSR expression, potentially influencing hormone therapy responses. However, Busic-Pavlek et al. [2] found a negative correlation (*p* = 0.033) between ER and CaSR expression, suggesting that tumors with higher ER levels may downregulate CaSR expression. However, our study identified no significant correlation between these two markers, indicating that the interplay between them may be influenced by additional molecular determinants or patient-specific factors.

Interestingly, our findings demonstrated a strong positive correlation between CaSR and both HER2 and BRCA1 gene expressions (*p* = 0.001 for both). These results are in alignment with previous research [2, 20] which also reported a positive association between CaSR and HER2 expression. Additionally, other studies have suggested that BRCA1 loss leads to a downregulation of CaSR expression [21]. Given that HER2 and BRCA1 are key regulators of tumor progression, their loss in certain breast cancer subtypes may result in reduced CaSR expression, reinforcing the notion that CaSR could act as a tumor suppressor under specific conditions.

Collectively, these findings highlight the context-dependent nature of CaSR’s role in breast cancer, emphasizing the need for further investigation into its mechanistic interactions with key molecular pathways. Understanding these relationships could provide insights into CaSR’s potential as a prognostic biomarker or therapeutic target in breast cancer management.

### CaSR and oncogenic MAPK and AKT signaling pathways interplay

Our findings revealed a strong positive correlation between CaSR expression and the activation of both p38 MAPK and AKT signaling pathways (*p* = 0.001 for both genes). This association suggests a potential role for CaSR in modulating key intracellular signaling cascades that drive tumor progression, survival, and metastasis.

Previous studies have established mechanistic links between CaSR activation and oncogenic signaling pathways. For instance, Orduña-Castillo et al. [22] demonstrated that CaSR indirectly enhances AKT phosphorylation in metastatic prostate cancer cells, thereby promoting cell proliferation and migration. Similarly, Chavez-Abiega et al. [5] reported that CaSR activation can stimulate multiple protein kinase families, including MAPKs and AKT, reinforcing its role in tumor cell survival, invasion, and therapy resistance.

The MAPK and AKT pathways are central regulators of cellular growth, apoptosis evasion, and metastasis [23, 24]. Their hyperactivation has been implicated in various aggressive cancer subtypes, making them attractive therapeutic targets. The observed correlation between CaSR expression and these pathways suggests that CaSR may serve as an upstream regulator, potentially influencing tumor aggressiveness and response to treatment. Given the oncogenic potential of these pathways, targeting CaSR-mediated MAPK/AKT signaling could offer new therapeutic opportunities. For instance, inhibitors targeting CaSR activity or downstream kinases may help suppress tumor growth and metastasis, particularly in cancers where CaSR functions as an oncogene. However, considering its dual role in different cancer types, further research is needed to determine whether CaSR inhibition would be beneficial or detrimental in specific tumor contexts.

### Unveiling the link between CaSR and cellular adhesion pathways in breast cancer

Cell-cell adhesion is a fundamental process in maintaining tissue architecture, and its disruption is a hallmark of cancer progression [25, 26]. While classical cadherins, such as E-cadherin, have been extensively studied in the context of tumor adhesion and metastasis, desmosomal proteins are emerging as critical players in cellular cohesion and signaling pathways [27, 28].

Our findings reveal a novel and specific association between CaSR and desmosomal proteins DSG1 and DSC1, rather than classical E-cadherin-mediated adhesion or β-catenin expression, indicating an alternative CaSR-dependent pathway influencing cell-cell cohesion and metastatic potential in breast cancer. These data contrast with studies in colon cancer, where CaSR has been shown to enhance E-cadherin expression and suppress the Wnt/β-catenin signaling pathway [29, 30].

The biological significance of CaSR in regulating desmosomal proteins has not been previously explored in breast cancer. Desmosomes play a key role in epithelial integrity and mechanical resistance, and alterations in DSG1 and DSC1 have been implicated in cancer cell migration and invasion [31]. Given the well-established role of CaSR in calcium homeostasis and signaling, our findings raise the possibility that CaSR may regulate tumor progression by reinforcing desmosomal adhesion rather than acting through classical cadherin-mediated pathways.

### Exploring the role of CaSR in protein trafficking and metastasis

Protein trafficking is a critical process in cancer cell survival, signaling, and metastasis [32]. CaSR has emerged as a key regulator of cellular processes beyond calcium homeostasis, including protein transport, receptor recycling, and intracellular trafficking.

Recent CaSR interactome analyses [1, 4] have identified 94 novel interacting proteins involved in mitochondrial, Golgi, plasma membrane, and endoplasmic reticulum processing, along with pathways linked to transport, trafficking, endocytosis, and degradation. These findings suggest a broader functional role for CaSR in cellular dynamics and cancer progression.

Interestingly, Orduña-Castillo et al. [22] proposed that CaSR activation promotes secretory pathways, potentially facilitating the release of pro-tumorigenic factors and enhancing metastatic potential in cancer cells. However, despite its known role in endocytosis and exocytosis, our study found no correlation between CaSR expression and key genes involved in these processes, including Clathrin, Cav-1, DYN1, DYN2, DYN3, and Beclin 1.

One exception remains Cav-1, a component of caveolae-specialized membrane microdomains involved in signal transduction and vesicular trafficking. Tian et al. [4] reported that Cav-1 facilitates CaSR colocalization with signaling molecules, suggesting that Cav-1-dependent pathways could be a crucial mediator of CaSR’s effects on trafficking. Further studies are necessary to determine whether CaSR utilizes caveolar endocytosis for receptor recycling, intracellular signaling, or metastatic regulation [5].

### CaSR expression patterns and prognostic significance in breast cancer

Our immunohistochemical analysis of CaSR expression across different breast cancer grades and fibroadenoma tissues provides critical insights into its potential role in tumor progression and prognosis. Moderate cytoplasmic staining (+ 2) was observed in fibroadenoma tissues, serving as a control, while strong membranous and cytoplasmic staining (+ 3) was detected in grade II tumors without metastasis or lymph node invasion. Interestingly, as the tumors became more aggressive, CaSR expression decreased, with weak cytoplasmic staining (+ 1) in grade II/III tumors with metastases and lymph node invasion and no detectable staining in grade III tumors with metastasis but no lymph node involvement.

These findings align with previous studies by Molostvov et al. [33] and Xi et al. [34], which suggested that cytoplasmic CaSR localization may be influenced by factors such as high receptor synthesis, post-translational modifications, or receptor recycling mechanisms. The observed pattern of high CaSR expression in lower-grade tumors and its reduction in more aggressive, metastatic cancers suggests a potential link between CaSR downregulation and tumor progression. This may indicate that while CaSR plays a role in early-stage tumor growth, its loss could contribute to enhanced metastatic potential and poorer prognosis in breast cancer.

Further investigations are necessary to clarify whether CaSR downregulation is a consequence of tumor dedifferentiation, a shift in cellular signaling pathways, or an adaptive mechanism favoring metastasis. Understanding these mechanisms could pave the way for utilizing CaSR as a prognostic marker or a therapeutic target in breast cancer management.

### Future directions and therapeutic potential

Our findings reveal a novel regulatory network involving CaSR and non-coding RNAs, shedding light on unexplored mechanisms of CaSR modulation in breast cancer. The positive correlations between CaSR and HOTAIR (*p* = 0.016), BC200 (*p* = 0.001), and SNOR78 (*p* = 0.001) suggest that lncRNAs and snRNAs may influence CaSR expression and function, a relationship that has not been previously reported. Such associations suggest that CaSR may be modulated by epigenetic and post-transcriptional mechanisms during tumor progression. These interactions warrant further investigation into how non-coding RNAs regulate CaSR-mediated signaling.

Despite the strengths of this study, several limitations should be acknowledged. First, the relatively limited sample size and the retrospective design may restrict the generalizability of the findings and preclude definitive causal inferences. Second, while significant correlations between CaSR expression and oncogenic signaling pathways, non-coding RNAs, and adhesion-related molecules were identified, functional assays were not performed to directly validate the mechanistic roles of CaSR in regulating these pathways. Third, immunohistochemical analysis was conducted on a limited number of tissue samples per tumor grade, which may not fully capture intratumoral heterogeneity. Fourth, although CaSR is classically described as a membrane receptor, nuclear CaSR immunoreactivity was observed with no functional or mechanistic validation to assess its biological significance. Therefore, the nuclear CaSR signal observed here should be interpreted with caution and regarded as an exploratory finding that requires further investigation. Finally, the lack of longitudinal follow-up and survival data prevented evaluation of the prognostic value of CaSR expression.

By integrating gene expression profiling, clinicopathological correlations, and protein localization analyses, this study advances current knowledge by positioning CaSR as a context-dependent regulator of breast cancer progression with potential prognostic and therapeutic relevance. However, to substantiate CaSR as a reliable biomarker or therapeutic target, future studies in larger patient cohorts are warranted, with particular emphasis on deciphering its role in MAPK/AKT-driven cancer pathways. Key areas of exploration include: (i) Mechanistic insights by understanding how CaSR modulates MAPK and AKT activation in various cancer subtypes; (ii) Therapeutic implications by assessing whether CaSR expression affects responses to MAPK/AKT inhibitors and identifying patients who might benefit from CaSR-targeted therapies; (iii) CaSR in cellular adhesion and trafficking by investigating CaSR’s interaction with desmosomal proteins (DSG1, DSC1) and its involvement in caveolin-mediated trafficking, which may contribute to tumor invasiveness.

By addressing these critical questions, future research can determine whether CaSR serves as a key regulator of oncogenic signaling and metastasis, ultimately paving the way for more personalized and effective breast cancer treatments.

## Supplementary Information

Supplementary Material 1.

## Data Availability

All data generated and analyzed in this study are included in this article.
