# Supplementary material for: Modulated calcium-sensing receptor (CaSR) expression in human breast cancer provided insights into tumor progression and therapeutic potential
Source: J Egypt Natl Canc Inst. 2026 Jan 19;38:1. doi: 10.1186/s43046-026-00338-x (PMC13262382; doi:10.1186/s43046-026-00338-x)
Supplement: Supplementary file 1 — Supplementary Material 1. [file 43046_2026_338_MOESM1_ESM.docx]

**Table S1:** Clinicopathological Prognostic Parameters of Invasive Ductal Carcinoma (IDC) in Breast Cancer Patients

| **Parameters** | | **Number (%)** |
| --- | --- | --- |
| **Age (years)** | Min-Max | 30-77 |
|  | Mean ± SD | 52.8 ± 12.7 |
|  | Median age | 50.5 |
| **Menopausal status** | Pre | 27 (38.7) |
|  | Post | 43 (61.3) |
| **Tumor size (T) (cm)** | Min – Max | 1- 9 |
|  | <2 | 10 (14.3) |
|  | 2-4 | 45 (64.3) |
|  | >4 | 15 (21.4) |
| **Tumor grade** | II | 56 (80) |
|  | III | 14 (20 ) |
| **Vascular invasion** | Negative | 2 (2.9) |
|  | Positive | 68 (97.1) |
| **Lymph node metastasis** | Negative | 24 (34.3) |
|  | Positive | 46 (65.7) |
| **ERα** | Negative (0) | 9 (12.9) |
|  | Positive | 61 (87.1) |
|  | +1 | 22 (31.4) |
|  | +2 | 28 (40) |
|  | +3 | 11 (15.7) |
| **HER2** | Negative (0) | 31 (44.3) |
|  | Positive | 39 (55.8) |
|  | +1 | 23 (32.9) |
|  | +2 | 7 (10) |
|  | +3 | 9 (12.8) |
